# SPECT-CT: Technology trail

**DOI:** 10.4103/0972-3919.72683

**Published:** 2010

**Authors:** Anshu Rajnish Sharma

**Affiliations:** Editor-in-Chief, IJNM Consultant, Nuclear Medicine & PET Kokilaben Dhirubhai Ambani Hospital and Medical Research Institute, Four Bunglows Andheri (W), Mumbai - 400 053, India. Email: anshurajneesh@rediffmail.com

Dear Readers,

Use of computed tomography (CT) in nuclear medicine is a reality now. Rapid growth of positron emission tomography–computed tomography (PET–CT) in the present decade proves its clinical utility and acceptability in comparison to any upcoming imaging modality. It has revolutionized the nuclear medicine practice tremendously and made its impact in oncology. In the last 6 years, more than 50 PET–CTs have been installed in India since the inception of first PET–CT at Tata Memorial Centre, Mumbai. Industry is expecting more growth in this segment as requirement and patient load are increasing.

But integration of CT to gamma camera, SPECT–CT, is not showing similar dream run. Number of SPECT–CT scanners is about half as compared to PET–CT installations in India. In 2009, annual growth rate was less than 10% in conventional nuclear medicine segment according to industry reports. Why does this lag exist in acceptability for SPECT–CT? Is SPECT–CT not clinically relevant or are nuclear medicine physicians not accepting this change in conventional configuration? Are other forces or reasons creating resistance?

Let us analyze the situation in an unbiased manner and critically. Is SPECT–CT more useful than gamma camera (SPECT) in routine clinical indications of radionuclide imaging? In the background of enough scientific evidence and adequate clinical experience, I personally do not feel that integration of CT with SPECT gamma camera will have a clinically significant impact and contribution in acquisition and interpretation of routine nuclear medicine procedures like DTPA/EC renal scans or DMSA renal scans, hepatobiliary scans, thyroid scan including biphasic Tc-99m SesatMIBI parathyroid scintigraphy, colloid liver scintigraphy, bone scan and myocardial perfusion SPECT studies, which make majority of our routine clinical workload. But integration of CT (even low dose/low definition CT) with SPECT camera has the ability to enhance the image quality by means of doing attenuation and scatter correction. It is now an accepted fact that CT attenuation map provides best attenuation correction as compared to transmission maps in nuclear cardiology. According to American Society of Nuclear Cardiology guidelines, it is desirable to do attenuation correction for myocardial perfusion SPECT studies, if available. It improves the image quality and diagnostic accuracy of myocardial perfusion SPECT. So, CT integration with SPECT camera has value addition in nuclear cardiology, even if it is low resolution, low dose and low current CT tube. Therefore, SPECT–CT camera is desirable in those centers having dedicated Nuclear Cardiology unit and which believe in quality patient care.

For localization of bone pathologies on bone SPECT, adrenal tumors on MIBG scan, neuroendocrine tumors with ^111^In Octreotide, staging/restaging of differentiated thyroid cancer with diagnostic ^131^I radioiodine scan, the suboptimal image quality of anatomical map provided by these low dose integrated CT is undesirable and unacceptable due to poor resolution and prolonged acquisition time.

However, there is growing evidence in literature of proven clinical utility of recent generation SPECT–CT with diagnostic CT capabilities, in the endocrinology for the evaluation of Neural Crest and adrenal tumors with ^123/131^I-MIBG, neuroendocrine tumors with ^111^In-Octreotide, parathyroid adenoma with Tc-99m SestaMIBI, postoperative workup and dose determination of differentiated thyroid cancers with ^131^I radioiodine and localization of local nodal recurrence in prostate cancers with ^111^In Capromab pendetide (ProstScint). For all these indications, SPECT–CT can play a significant role as PET–CT is either not indicated or there is limited availability of Ge/Ga68 generators.

There is growing evidence in favor of use of SPECT–CT in gastroenterology for localization of occult lower gastrointestinal bleeding and bile leak. SPECT–CT has improved the confidence of interpreting inconclusive/indeterminate pathology demonstrated on bone scan and increased the specificity. With CT component of SPECT–CT, we are able to differentiate benign and malignant pathologies based on their location and morphological appearance.

The current generation high-end SPECT–CT (64 slices or more) can act as Single Stop Shop for evaluating a patient of coronary artery disease in a non-invasive manner by doing myocardial perfusion SPECT scan, attenuation correction for better image quality, coronary artery calcium scoring for estimation of global atherosclerotic burden and CT coronary angiography in a single sitting. The cardiac SPECT–CT image received SNM’s “Best Picture Award” for its innovative technology. In times to come, as evidence is growing, probably Tc-99m MAA Lung Perfusion SPECT–CT (CTPA; CT pulmonary angiography) may become the modality of choice for diagnosing pulmonary embolism. Therefore, this hybrid technology has a bright future and is going to play a significant role in patient management. But for this, we also need to understand that we are venturing into others’ territory and may experience conflict of interests. We need to prepare and train ourselves with rapidly advancing technology for providing quality patient care.

There are few understandable counterpoints against the justified utility of SPECT–CT. Usually, SPECT and nuclear medicine studies take quite longer acquisition time in comparison to CT component in integrated SPECT–CT machine. Can we afford to keep such a high-end CT component (64 slices or more) to remain idle during radionuclide scan acquisition? Don’t you think underutilization of such costly equipment component will be cost-effective? There is recent development in SPECT detector technology (CZT; cadmium zinc telluride). These semiconductor detectors are fast, sensitive, slim, give high resolution image and can catch up with the speed of CT acquisition.

I personally feel that SPECT–CT like the PET-CT will have more convincing communication with our referring physicians as there will be more credible functional information verified by position and location due to the available morphological background.

For this credible information, we will require extra space, extra shielding for the machine, extra cost and more training and learning experience with the goal to keep radiation exposure as low as reasonably achievable.

